# Case Report: Very Late, Atypical Extra-Medullary Relapse in a Patient With Acute Promyelocytic Leukemia (APL) Rescued With a Transplant-Free Approach

**DOI:** 10.3389/fonc.2021.699886

**Published:** 2021-06-29

**Authors:** Matteo Molica, Carla Mazzone, Tiziana Ottone, Pasquale Niscola, Elisabetta Abruzzese, Stefano Fratoni, Maria Teresa Voso, Paolo de Fabritiis

**Affiliations:** ^1^ Haematology Unit, St. Eugenio Hospital, Rome, Italy; ^2^ Department of Biomedicine and Prevention, University Tor Vergata, Rome, Italy; ^3^ Surgical Pathology, Hematopathology Unit, St. Eugenio Hospital, Rome, Italy

**Keywords:** acute promyelocitic leukemia, transplant free approach, bcr3 variant, all-trans retinoic acid and arsenic trioxide combination treatment, very late relapse

## Abstract

Relapses of acute promyelocytic leukemia (APL) beyond 7 years from the first molecular remission are exceptional, and it is unclear whether these relapses represent a new, therapy-related leukemia rather than a delayed relapse of the original leukemic clone. The increase extra-medullary relapses (ER) in the era of all-trans retinoic acid (ATRA) therapy suggests a potential correlation between ATRA therapy and ER, and several potential explanations have been proposed. The gold standard post-remission approach, particularly for patients in late relapse, has not yet been established. The benefit of a transplant approach has been questioned in this setting because continuing ATRA-arsenic trioxide (ATO) might be curative. Here we report on the case of an APL patient who relapsed 9 years after achieving her first molecular complete remission (mCR) and who showed an atypical isolated localization at nodal sites, including the into- and peri-parotid glands. Genomic *PML/RARa* breakpoint analysis detected the same bcr3 *PML/RARa* hybrid gene in DNA purified from bone marrow and lymph nodes, suggesting that the relapse was because of the reemergence of the initial clone. This case shows that APL, treated with ATRA and cytotoxic drugs, may still emerge in extra-medullary sites even after a very prolonged mCR and could be salvaged with an ATO-based protocol, not including a transplant approach.

## Case Report

Late relapses in acute promyelocytic leukemia (APL) patients that occur three or more years from the achievement of complete remission (CR) are very rare, and relapses beyond 7 years from the initial diagnosis are exceptional. A few cases of late APL relapses treated with all-trans retinoic acid (ATRA) in combination with other approaches have been reported (1–10). Most late relapses are the result of the identical immunophenotypic, cytogenetic, and molecular features already present at diagnosis, suggesting that relapse emerged through the initial leukemic clone (3–5). Approximately 3% to 5% of adult APL presents an extra-medullary relapse (ER) (11, 12). The incidence of ER, which has risen in the era of ATRA therapy, suggests a potential correlation between ATRA therapy and ER (13). Two possible speculative reasons have been contemplated: 1) an increased infiltration of APL leukemic blasts into sanctuary sites because of the effect of ATRA on adhesion molecules and 2) prolonged disease-free survival in treated APL patients (13). However, the main reason is that, in ATRA era, the rate of long-term survivors has increased exponentially, giving far more opportunities to develop late relapses than in the pre-ATRA era. Here we report on the case of an APL patient, treated using the GIMEMA AIDA 2000 protocol, who relapsed 9 years after achieving her first molecular complete remission (mCR) and who showed an atypical presentation at nodal sites into- and peri-parotid gland.

A 43-year-old female was diagnosed with classic APL in March 2011. At diagnosis, peripheral blood count showed WBC 3.6 × 10^9^/l with 70% atypical promyelocytes, platelets 15 × 10^9^/l, and Hb 12.1 g/dl. The immunophenotypic pattern (CD13+, CD33+, HLA-DR−], karyotypic evaluation [t(15;17) as unique abnormality), and molecular analysis (positivity for *PML/RARa* bcr3) were consistent with APL. The patient was enrolled into the AIDA 2000 protocol and achieved CR following induction with ATRA plus idarubicin (IDA). Molecular remission was achieved after the first consolidation course, and treatment was discontinued in October 2013, after three consolidation cycles and 2 years of maintenance therapy based on oral 6-mercaptopurine (50 mg/m^2^) and intramuscular methotrexate (15 mg/m^2^) alternating with oral ATRA for 15 days every 3 months. The patient remained in CR^MRD−^ for 9 years. In May 2020, the patient presented with a solid mass in the parotid region: an ultrasound and a computed tomography (CT) scan of the neck showed the presence of four right intra-parotid lymph nodes (maximum diameter 2.5 cm) associated with sub-centimetric peri-parotid, sub-maxillary, and retropharyngeal lymph nodes. The bone marrow was morphologically in CR; however, a molecular relapse of the original bcr3 *PML/RARa* rearrangement was detectable by RT-PCR.

**Figure 1:**
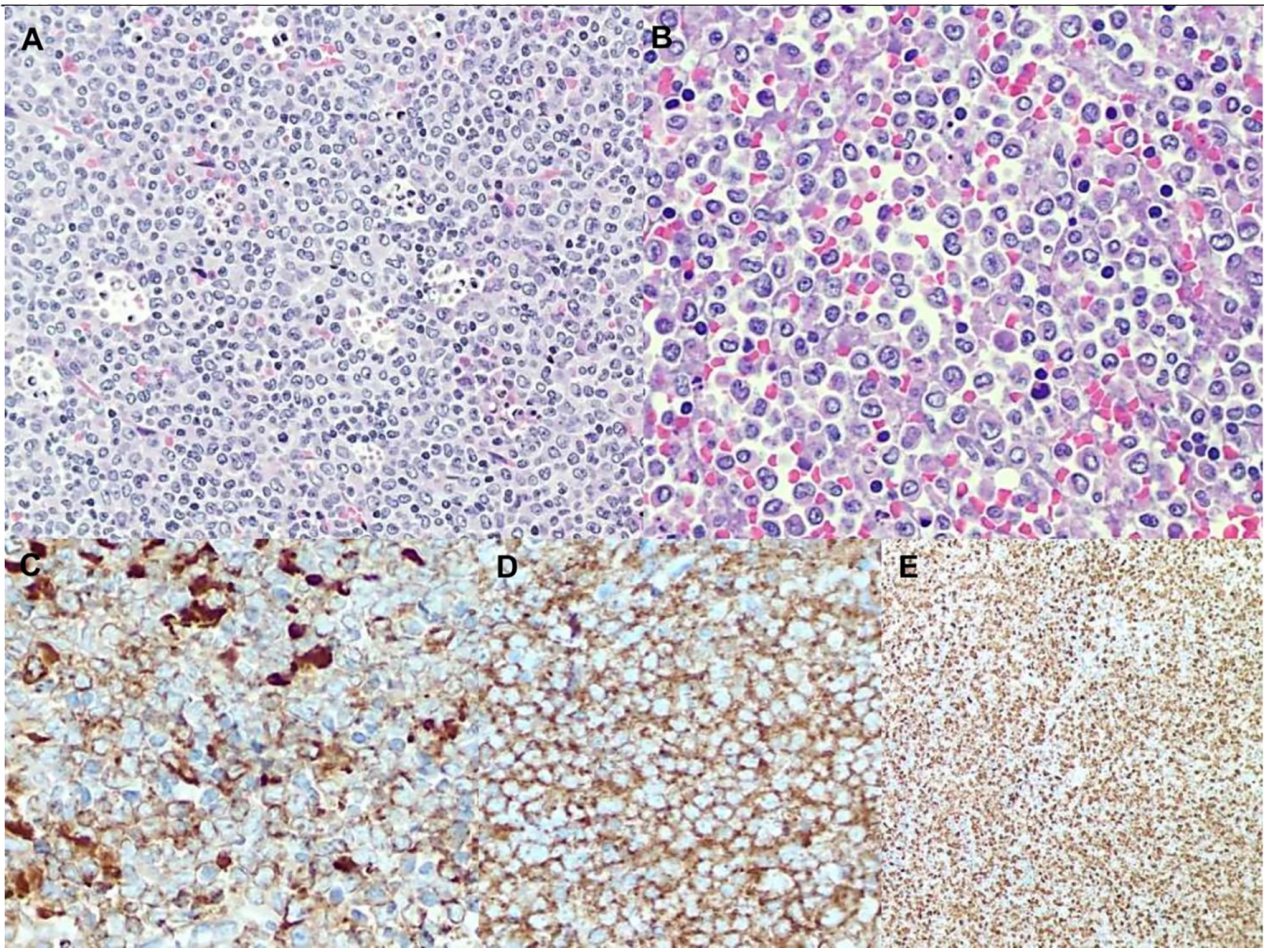
**(A)** low magnification shows a blastic population with diffuse pattern of growth admixed with a lot of tingible body macrophages and apoptotic debris. **(B)** High magnification reveals blastic immature promyelocytes with hypergranular eosinophilic cytoplasm, kidney-shaped or lobed nuclei and prominent central nucleoli. Immunohistochemistry shows a diffuse and strong expression of both MPO **(C)** and CD33 **(D)** along with very high proliferation index Ki67 **(E)**.

**Figure 2:**
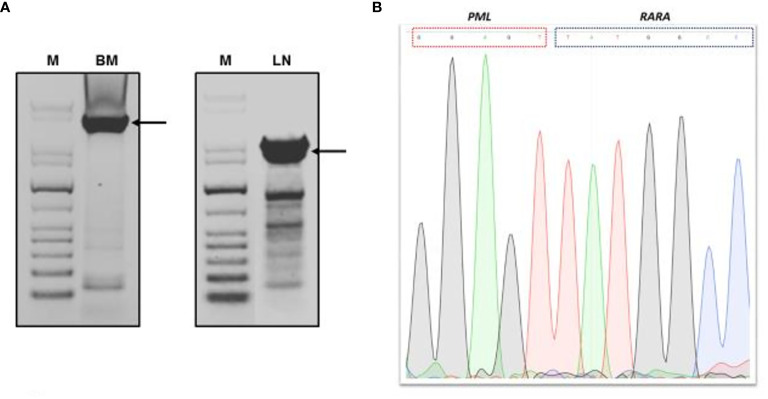
Genomic *PML/RARA* breakpoint analysis by long-range nested PCR. **(A)** Agarose gel electrophoresis of long-range PCR products from bone marrow and lymph node section DNA analysis. **(B)** Sanger sequencing result of long-range PCR product of *PML/RARA* genomic amplification derived from BM analysis. Sequence was aligned to the intronic sequences of *PML* and *RARa* genes. M, GenLadder 1kb DNA Ladder; BM, bone marrow; LN, lymph node. Arrows indicated the long-range PCR products purified from gel.

A biopsy of an intra-parotid lymph node was performed. Histopathological examination revealed a lymph node architecture totally effaced by a massive population of atypical promyelocytes with kidney-shaped and/or irregular lobed nuclei, prominent central nucleoli, and an eosinophilic cytoplasm that was hypergranulated. Immunohistochemistry revealed a diffuse and strong expression of both MPO and CD33, whereas expression of CD117, CD34, CD68RPGM1, CD14, CD13, CD163, CD56, PAX5, CD3, and large-spectrum cytokeratin AE1/AE3 was absent. Molecular analysis of embedded paraffin tissue showed the presence of the t(15;17) *PML-RARa* fusion gene, confirming the final diagnosis of APL, also defined as a granulocytic sarcoma promyelocytic type, because of its own extramedullary nodal localization (**Figure 1**). To determine the exact chromosomal breakpoint position in the *PML* and *RARa* genes, long-range PCR was performed on DNA samples derived from bone marrow (BM) MNC and the lymph node biopsy. The *PML/RARa* bcr3 isoform was detected using nested real-time polymerase chain reaction (RT-PCR) on the BM sample collected at relapse. DNA extracted from BM-MNC and the lymph node biopsy was also analyzed using long-range PCR. Using different primers combinations, we confirmed the presence of the *PML/RARa* hybrid that was detected in DNA purified from the lymph node sections (**Figure 2**). Sanger sequencing of both PCR products showed the same breakpoints locations in the *PML* and *RARa* genes, at nucleotide position 996 of the PML intron 3 and position 14392 of the *RARa* intron 2 (**Figure 2**). The breakpoint locations were the same as in the original samples harvested at the time of initial APL diagnosis, in 2011.

The patient was treated with an induction treatment based on the following drugs combination: IDA 12 mg/m^2^ on days 1 and 3, arsenic trioxide (ATO) 0.15 mg/kg from day 5 to day 28, and ATRA 45 mg/m^2^ from day 1 to day 28). A second mCR, assessed by RT-PCR of the *PML/RARa* hybrid, and the complete disappearance of lymph node involvement from a CT-scan were determined after the induction course. According to the radiological response after induction, located radiotherapy on lymph nodes, which was contemplated among potential treatments at relapse, was not performed. ATO and ATRA treatments were continued with three further consolidation courses given at monthly and bi-weekly intervals, respectively. Four doses of intrathecal cytarabine were administered during consolidation. She was closely monitored by RT-PCR throughout the treatment and she will continue to be assessed every two months for at least two years. The patient remains in continuous second mCR until last follow up (May 2021) leading a normal life. To our knowledge, this is one of the latest relapses observed in an APL patient treated with ATRA plus chemotherapy (1, 3, 7). The unusual aspects of this case appear to be due to two main reasons: a relapse after a prolonged period of documented mCR (9 years), and the atypical site of extra-medullary disease. Molecular relapse in this patient was associated with an intra-parotid lymph node involvement, a site infrequently reported in APL relapse and usually present in earlier disease recurrence (3, 14). Genomic *PML/RARa* breakpoint analysis by RT-PCR detected the same bcr3 *PML/RAR*a hybrid gene in DNA purified from BM and lymph nodes, suggesting that the relapse was due to reemergence of the initial clone. Whereas central nervous system and skin involvement in APL relapse have been associated with mechanisms mediated by cellular adhesion molecules (CD56, LFA-1, and VLA-4) probably over-expressed in response to ATRA-driven differentiation (15, 16), the issue as to whether ATRA promotes nodal involvement in APL relapses is still unknown. Because patients affected by ATRA syndrome have APL cells that have infiltrated multiple tissues and organs, it has been hypothesized that ATRA could promote the migration of differentiating blasts into several tissues. These blasts could form a reservoir of viable leukemic cells that might later proliferate and result in an extra-medullary recurrence (17, 18). Our patient achieved a second mCR and extra-medullary response after the induction course and three further consolidation courses based on ATO and ATRA combination. Because of the prolonged mCR achieved after the initial treatment, no hematopoietic stem cell transplant (HSCT) option was offered to the patient. The gold standard post-remission approach, especially for late relapse patients, is not yet well established. A registry study of the European Leukemia Net, which analyzed 155 APL relapsed patients showed the efficacy of allogenic and autologous HSCT as a consolidation treatment for patients with early and late relapses who did not achieve a mCR (19).

Based on recent studies (19–23), autologous HSCT should be considered the first choice for eligible patients achieving second molecular remission. However, a recent NCRI report questions the role of transplantation, at least in patients achieving molecular remission with ATO and ATRA who do not have CNS disease at relapse and who have received a full course of consolidation with ATO (24).

However, the benefit of a transplant approach could be questioned in patients relapsing after a very prolonged first CR because continuing ATRA-ATO might in fact be curative. Limited data have been reported for patients who received prolonged ATRA/ATO therapy after a first relapse without a final consolidation with a stem cell transplant. A recent update of 22 patients indicated that only two patients underwent transplant and the rest received additional cycles of ATRA/ATO. The four-year overall survival probability was 85% with a disease-free survival rate of 74%, supporting the potentially curative effect of prolonged ATO treatment especially in patients with a long first mCR (25). In conclusion, this case shows that APL, treated with modern combination therapies including ATRA and cytotoxic drugs, may still emerge in extra-medullary sites even after a very prolonged molecular remission (9 years) and could be salvaged with an ATO-based protocol not including a HSCT.

## Data Availability Statement

The original contributions presented in the study are included in the article/supplementary material. Further inquiries can be directed to the corresponding author.

## Ethics Statement

Written informed consent was obtained from the patient for the publication of this case report and the accompanying images.

## Author Contributions

MM and PF designed the study and wrote the paper. MM, CM, EA, and PN followed the patient. TO and MV performed the molecular studies. SF made histopathological examination. PF supervised the study. All authors contributed to the article and approved the submitted version.

## Conflict of Interest

The authors declare that the research was conducted in the absence of any commercial or financial relationships that could be construed as a potential conflict of interest.
